# Professional Performance of Assistant Teachers Supporting Children With Autism in the United Arab Emirates: Mixed Methods Observational Study

**DOI:** 10.2196/86784

**Published:** 2026-09-21

**Authors:** Fawaz Habbal, Abdulla Abdulalee Alhumaidan, Hajar Aljaberi, Fadi Nayef Bani Mostafa, Suzan Almali, Afaf Almenhali

**Affiliations:** 1 Emirates Scholar Center for Research and Studies, F9MG+XGH, Al Falah St - Al Danah - Zone 1, Abu Dhabi, PO Box 28686, United Arab Emirates, 971 24093159; 2 Zayed Authority for People of Determination, Abu Dhabi, United Arab Emirates; 3 National Academy for Child Development, Abu Dhabi, United Arab Emirates

**Keywords:** assistant teachers, autism spectrum disorder, inclusive education, mixed methods study, classroom observation, parent engagement, teacher competency, program evaluation, diploma training, special education, classroom practice, parent-teacher meetings, observational assessment, cultural responsiveness, professional development, individualized education plan–aligned practice, IEP-aligned practice, autism support

## Abstract

**Background:**

Assistant teachers play a central role in supporting children with autism spectrum disorder in United Arab Emirates inclusive classrooms. Despite this importance, no prior study has used validated observational instruments to systematically evaluate assistant teachers’ competency profiles across classroom practice and parent interaction domains simultaneously in this cultural context.

**Objective:**

This program evaluation study assessed the professional performance of assistant teachers supporting children with autism spectrum disorder, examining classroom instructional competencies and parental engagement behaviors within a single United Arab Emirates diploma training program.

**Methods:**

A convergent mixed methods design was used. Ten assistant teachers who completed a national diploma training program were assessed by 3 independent expert evaluators across 50 site visits (n=25, 50% classroom observations; n=25, 50% parent meeting observations) conducted in January 2025 and March 2025. Tool 1 assessed 11 standardized classroom competency criteria, and tool 2 assessed 8 parent interaction criteria, both using a 5-point Likert scale. Mean scores were calculated by averaging all evaluator ratings across observation sessions per criterion. Internal consistency was assessed using the Guttman λ (λ=0.79 for tool 2). Formal interrater reliability was not computed; consensus-based resolution was used in cases in which evaluator ratings differed, which is acknowledged as a methodological limitation. Qualitative field notes were analyzed using the 6-phase thematic analysis by Braun and Clarke.

**Results:**

Classroom performance means were 4.56 (SD 0.19; very high) in phase 1 (January 2025) and 4.55 (SD 0.23; very high) in phase 2 (March 2025). All teachers (10/10, 100%) achieved ceiling scores for daily living skill support and cultural adherence (mean 5.00, SD 0.00) across both phases. Safety and hygiene criteria scores were in the high range (mean 4.00, SD 0.00 to 4.10, SD 0.32). In parent interaction assessments (tool 2), the overall mean was 4.75 (SD 0.20; very high). The highest-ranked criteria were empathetic family treatment (mean 4.92, SD 0.28) and meeting preparation (mean 4.88, SD 0.33). The lowest-ranked criterion was clarity of structured communication (mean 4.48, SD 0.51; very high). Qualitative themes identified culturally responsive communication, emotional attunement, and individualized education plan–aligned practice. Repeated SD values of 0.00 and the absence of formal interrater reliability statistics are limitations that should be considered when interpreting these findings.

**Conclusions:**

Within this program-specific evaluation, assistant teachers demonstrated consistently high professional competence. These preliminary findings suggest that diploma-based training can support culturally aligned and technically proficient inclusive practice. Targeted professional development in proactive safety and hygiene integration and structured individualized education plan–linked parent communication are recommended. Replication with larger, multi-institutional samples is required before broader conclusions can be drawn.

## Introduction

### Background and Rationale

Autism spectrum disorder (ASD) presents a heterogeneous constellation of behavioral, communicative, and social challenges requiring individualized and responsive educational approaches. In the United Arab Emirates (UAE), national policy has progressively mandated equitable access to inclusive education for all children with disabilities, yet the pedagogical workforce supporting this mandate remains underexamined. Assistant teachers serve as the primary point of daily contact between children with ASD and the broader educational environment, occupying a strategically critical but often underresearched role [1,2]. They collaborate with lead educators to implement individualized education plans (IEPs), manage behavior, provide adaptive learning support, and facilitate daily routine transitions.

Despite their centrality, assistant teachers are frequently absent from formal performance evaluation frameworks in the region. Prior research has focused predominantly on lead teachers or on Western educational contexts where cultural and organizational structures differ substantially from those in the UAE [3,4]. Recent work by Opoku et al [5] and Benson and Perepa [6] has begun to document the expanding roles and training needs of teaching assistants in Gulf Cooperation Council contexts; however, no study to date has applied validated observational instruments to assess competency profiles across both classroom practice and parent interaction domains simultaneously in this setting. The systemic dimension of inclusive education is also relevant: Mahmood et al [7] demonstrate that teacher and administrator support together shape the adaptation of students with special needs, underscoring that evaluating teacher competencies in isolation provides only a partial picture of inclusive practice quality.

### Theoretical Framework: Culturally Responsive Pedagogy in the UAE Context

This study is informed by the framework of culturally responsive pedagogy (CRP), which holds that effective teaching requires active integration of students’ and families’ cultural values, linguistic practices, and community norms into instructional design and relational interactions [8,9]. Gay [8] identifies cultural competence, cultural communication, and inclusive curriculum as the 3 core dimensions of CRP, arguing that educators who embed cultural knowledge into practice produce stronger academic and social outcomes for culturally diverse learners. In the UAE context, CRP takes a distinctive form: assistant teachers are professionally expected to use Modern Standard Arabic (Fusha Arabic)—the formal, standardized register of Arabic used in education, religious practice, and official communication, distinct from regional colloquial dialects (*ammiyya*)—as the instructional medium; embody Islamic behavioral norms, including modest dress, respectful greetings (such as *As-salamu alaykum* [“Peace be upon you”]), and the integration of faith-based values into classroom routines; and serve as cultural mediators between school and family environments [10]. These expectations are not informal customs but formally incorporated into UAE educational policy [10] and into the diploma training program evaluated in this study. Ladson-Billings [9] argues that such culturally embedded practice is not supplementary but foundational to student belonging and learning efficacy, particularly for children with disabilities who are additionally vulnerable to social marginalization. This theoretical lens situates the study’s findings within a broader international framework, enabling comparisons across culturally specific inclusive education contexts beyond the UAE.

### Aims

This study was a program-specific evaluation, not a representative study of UAE assistant teachers broadly. It aimed to (1) document the overall and domain-specific professional performance of assistant teachers within 1 UAE diploma training program, (2) identify areas of consistent strength and relative development need within this sample, and (3) derive practice-based recommendations for training program improvement.

Three research questions guided the evaluation: (1) what were the overall and domain-specific professional performance levels of assistant teachers as assessed through structured observational tools? (2) How did assistant teachers demonstrate alignment with Emirati cultural and Islamic values, and what behavioral forms did this alignment take? (3) What practice-level gaps in safety, hygiene, and parent communication are indicated by these evaluation findings, and what training responses are appropriate?

## Methods

### Study Design

A convergent mixed methods design was used [11] combining structured quantitative observational ratings with concurrent qualitative field notes. Quantitative data provided standardized performance measurement across predefined criteria; qualitative data provided behavioral depth, contextual nuance, and interpretive triangulation. Both data streams were analyzed independently and subsequently integrated at the interpretation stage.

Observational data collection was conducted in January 2025 and March 2025 (phases 1 and 2, respectively) as part of a formal quality assurance evaluation initiated by the Zayed Authority for People of Determination in collaboration with the National Academy for Childhood Development. The diploma program itself had been developed and delivered over the preceding years; this evaluation assessed graduates at 2 time points approximately 2 months apart.

### Participants and Sampling

Participants were 10 assistant teachers who had recently completed the National Academy for Childhood Development’s assistant teacher diploma program in early childhood education and were actively employed in settings serving children with ASD. Purposive sampling was used to select a theoretically homogeneous posttraining sample. The sample of 10 is appropriate for the intensive, multi-visit observational design used in which each participant was assessed across multiple sessions by 3 evaluators, yielding 50 total site visits. However, given that all participants were drawn from a single diploma program, these findings must be understood as program specific and not generalizable to UAE assistant teachers broadly. Three independent expert evaluators, each with formal special education qualifications and experience in ASD-inclusive settings, conducted all observations.

### Instrumentation

Two purpose-built observational rubrics were developed and validated for this evaluation. Tool 1 assessed 11 standardized performance criteria covering classroom management, student supervision, transition monitoring, preparation of materials, daily living skill support, health and nutrition knowledge, safety and risk management, hygiene practices, emotional engagement, and cultural adherence. Tool 2 assessed 8 criteria for parent interaction: trust building, active listening and shared decision-making, meeting preparation, communication clarity, empathetic engagement, IEP-aligned guidance, professional communication style, and cultural sensitivity. The identical 11-criterion wording was used across both observation phases to ensure measurement consistency. Both instruments used a 5-point Likert scale (1=“strongly disagree”; 5=“strongly agree”). Content validity was established through review by a panel of 3 special education specialists. Internal consistency for tool 2 was estimated using the Guttman λ (λ=0.79), indicating acceptable reliability for small-sample educational research [12]. Tool 1 reliability was assessed using the same approach, yielding comparable internal consistency.

### Data Collection Procedure

A total of 50 site visits were conducted: 25 (50%) classroom observation sessions and 25 (50%) parent meeting observation sessions. Each session was observed in its entirety. Observations were unannounced (ie, teachers were not informed of the specific evaluation criteria in advance) to reduce social desirability bias. Evaluators independently completed quantitative rubric ratings and open-ended qualitative field notes after each session.

### Data Analysis

#### Quantitative Analysis

Mean scores were calculated by averaging all evaluator ratings across all observation sessions per criterion (ie, the unit of analysis was individual observation sessions aggregated across teachers and evaluators). SDs reflect variability across these aggregated ratings. Performance was classified using the range-based framework in Table 1. Three criteria yielded an SD value of 0.00, indicating complete uniformity across all evaluators and sessions; while theoretically possible for behaviorally anchored competencies, this pattern is unusual in applied educational research and may alternatively reflect limited rubric sensitivity, observer-related score compression, or reactivity effects from being observed, as discussed further in the Discussion section. The substantially higher SD for criterion 6 in phase 2 (1.15) is examined as a criterion-specific finding in the Results and Discussion sections. Formal interrater reliability (intraclass correlation coefficient [ICC] or κ) was not computed: evaluator ratings were averaged per criterion per session, and discrepancies were resolved through structured consensus discussion. The absence of a formal agreement statistic is acknowledged as a methodological limitation that affects the interpretive confidence that can be placed in individual criterion scores.

**Table 1.** Performance level classification based on mean score ranges.

Score range	Performance level
1.00 to <1.80	Very low
1.80 to <2.60	Low
2.60 to <3.40	Moderate
3.40 to <4.20	High
4.20 to 5.00	Very high

#### Qualitative Analysis

Field notes were analyzed using the 6-phase thematic framework by Braun and Clarke [13]: (1) data familiarization, (2) initial code generation, (3) theme development, (4) theme review, (5) theme definition, and (6) report production. Triangulation between quantitative and qualitative datasets was conducted to assess convergence and identify discrepancies.

### Ethical Considerations

This study constituted a formal institutional performance evaluation conducted as part of a structured quality-assurance process within an established professional training program. It did not involve recruitment of human research participants, experimental intervention, or collection of sensitive personal data requiring ethics board oversight. The evaluation was initiated and authorized by the Zayed Higher Organization for People of Determination and the National Academy for Child Development under their respective institutional quality-assurance mandates. All participating assistant teachers provided institutional consent through their professional program enrollment agreements. No data pertaining to children, families, or identifiable individuals were collected or reported. In accordance with the research ethics policy of Emirates Scholar Center for Research & Studies, Abu Dhabi—which stipulates that internal program evaluations and posttraining competency assessments conducted within institutional quality-assurance frameworks are exempt from formal ethics board review—independent ethics approval was not sought for this evaluation. All data were handled confidentially and reported in aggregate form without individual identification.

## Results

### Performance Classification Framework

Table 1 presents the scoring rubric used to classify all performance means.

### Classroom Observations: Phase 1 (January 2025; Tool 1)

Table 2 presents mean scores and SDs for the 11 classroom performance criteria in phase 1. The overall mean was 4.56 (SD 0.19; very high). All teachers (10/10, 100%) achieved the maximum score for criteria 3 (transition monitoring), 5 (daily living skill support), and 11 (cultural adherence; mean 5.00, SD 0.00 for each). All teachers (10/10, 100%) scored in the high range for criteria 7 (safety knowledge), 8 (hazard-free environment), and 9 (hygiene; mean 4.00, SD 0.00; mean 4.00, SD 0.00; and mean 4.10, SD 0.32, respectively). The items with an SD of 0.00 indicate complete uniformity across all evaluators and sessions; possible interpretations are discussed in the Discussion section.

**Table 2.** Mean scores and SDs for classroom performance—phase 1 (January 2025).

Criterion number	Criterion description	Score (1-5), mean (SD)^a^	Rank	Rating
1	Assisted in classroom management to ensure a responsive learning environment.	4.60 (0.52)	7	Very high
2	Supervised students during structured activities (eg, reading, assignments, and motor therapy).	4.50 (0.53)	9	Very high
3	Closely monitored students’ entry to and exit from the classroom.	5.00 (0.00)^b^	1	Very high
4	Prepared and effectively used classroom tools and resources (eg, audiovisual aids).	4.80 (0.42)	4	Very high
5	Supported students in daily living skills (eg, eating, dressing, and toileting).	5.00 (0.00)^b^	1	Very high
6	Demonstrated knowledge in health and nutrition relevant to child development.	4.75 (0.46)	5	Very high
7	Displayed knowledge of safety and security procedures relevant to children.	4.00 (0.00)^b^	10	High
8	Maintained a physically safe learning environment based on best practices.	4.00 (0.00)^b^	10	High
9	Ensured classroom hygiene and sanitation to prevent infectious diseases.	4.10 (0.32)	9	High
10	Demonstrated enthusiasm and emotional presence while interacting with children.	4.70 (0.48)	6	Very high
11	Adhered to Emirati cultural values, customs, and Islamic principles at all times.	5.00 (0.00)^b^	1	Very high

a Overall mean 4.56 (SD 0.19; very high).

b SD of 0.00 indicates complete evaluator and session uniformity; possible interpretations include genuine ceiling-level performance, observer-related score compression, or limited rubric sensitivity at the upper end of the scale (see the Discussion section).

### Classroom Observations: Phase 2 (March 2025; Tool 1)

Table 3 presents phase 2 data using the same standardized criteria. The overall mean was 4.55 (SD 0.23; very high), virtually identical to that of phase 1 (mean 4.56, SD 0.19). All teachers (10/10, 100%) again achieved ceiling scores for criteria 5 and 11 (mean 5.00, SD 0.00 each). Safety and hygiene criteria (7, 8, and 9) remained in the high range (mean 4.00, SD 0.00 in all cases), unchanged from phase 1. Criterion 6 (health and nutrition knowledge) showed the most substantive phase 2 change: the mean decreased from 4.75 (SD 0.46) to 4.33 (SD 1.15) but with a markedly higher SD. This criterion-specific increase in variability—against the background of otherwise compressed SDs across the dataset—is discussed separately in the Discussion section as a domain-specific finding rather than a general measurement issue.

**Table 3.** Mean scores and SDs for classroom performance—phase 2 (March 2025).

Criterion number	Criterion description	Score (1-5), mean (SD)^a^	Rank	Rating
1	Assisted in classroom management to ensure a responsive learning environment.	4.70 (0.48)	4	Very high
2	Supervised students during structured activities (eg, reading, assignments, and motor therapy).	4.70 (0.48)	4	Very high
3	Closely monitored students’ entry to and exit from the classroom.	4.60 (0.84)	7	Very high
4	Prepared and effectively used classroom tools and resources (eg, audiovisual aids).	4.80 (0.42)	3	Very high
5	Supported students in daily living skills (eg, eating, dressing, and toileting).	5.00 (0.00)^b^	1	Very high
6	Demonstrated knowledge in health and nutrition relevant to child development.	4.33 (1.15)^c^	8	Very high
7	Displayed knowledge of safety and security procedures relevant to children.	4.00 (0.00)^b^	9	High
8	Maintained a physically safe learning environment based on best practices.	4.00 (0.00)^b^	9	High
9	Ensured classroom hygiene and sanitation to prevent infectious diseases.	4.00 (0.00)^b^	9	High
10	Demonstrated enthusiasm and emotional presence while interacting with children.	4.70 (0.48)	4	Very high
11	Adhered to Emirati cultural values, customs, and Islamic principles at all times.	5.00 (0.00)^b^	1	Very high

a Overall mean 4.55 (SD 0.23; very high).

b SD of 0.00 indicates complete evaluator and session uniformity; possible interpretations include genuine ceiling-level performance, observer-related score compression, or limited rubric sensitivity at the upper end of the scale (see the Discussion section).

c SD of 1.15 indicates substantially higher interindividual variability in health and nutrition knowledge application; this criterion-specific finding is discussed in the Discussion section.

### Comparative Analysis: Phases 1 and 2

The near-identical overall means across phases (4.56, SD 0.19 vs 4.55, SD 0.23) indicate performance stability at the aggregate level over the 2-month interval. The identical domain strength pattern (daily living skills and cultural adherence) and development pattern (safety and hygiene) across both phases confirms that these represent consistent rather than transient characteristics within this sample. The sole criterion-level cross-phase difference was the increased SD for criterion 6 in phase 2, which is examined further below.

### Qualitative Findings: Classroom Observations

Table 4 presents the key behavioral patterns identified through thematic analysis of classroom field notes. Modern Standard Arabic (Fusha Arabic)—the formal register of Arabic used in UAE education and official discourse, distinct from colloquial spoken dialects—was universally used for instruction, reflecting the program’s explicit linguistic and cultural expectations.

**Table 4.** Key observed behavioral patterns during classroom visits—thematic summary.

Criterion number	Competency area	Key observed practices
1	Classroom management	Gave clear instructions before each activity, used a calm tone with visual cues, and divided the class into small groups to reduce distraction.
2	Supervision of activities	Maintained constant presence, used varied materials (cards, blocks, and sensory tools), and coordinated effectively with the lead teacher.
3	Attendance and transitions	Strictly adhered to entry and exit procedures, accurately documented attendance, and guided children to their belongings in an orderly manner.
4	Preparation of materials	Used age- and ability-appropriate audiovisual tools and prepared all materials before each session.
5	Daily living skill support	Encouraged self-help behaviors (eating and dressing) through verbal reinforcement and provided individualized bathroom guidance respecting developmental level.
6	Health and nutrition awareness	Communicated handwashing advice using age-appropriate language and explained the nutritional value of foods during mealtime.
7	Safety knowledge	Avoided hazardous tools, prompted children to sit safely, and demonstrated basic environmental risk awareness during transitions.
8	Hazard-free environment	Organized pathways, maintained visual supervision, and used child-sized furniture with safe spacing between activity areas.
9	Hygiene and sanitation	Disinfected tables before activities, prompted handwashing multiple times daily, and cleaned tools and materials after use.
10	Emotional engagement	Maintained consistent eye contact and warm demeanor and used an encouraging tone and expressive gestures.
11	Cultural and religious sensitivity	Delivered instructions in Modern Standard Arabic (Fusha Arabic), promoted Islamic values and etiquette, and wore modest attire aligned with UAE^a^ professional norms.

a UAE: United Arab Emirates.

Qualitative data largely corroborated the quantitative profile. Assistant teachers consistently demonstrated culturally aligned, emotionally attuned, and instructionally organized practice. Regarding safety and hygiene, evaluators noted that, although basic safety behaviors were present, they appeared reactive rather than proactively embedded within structured daily routines. This behavioral distinction is not fully captured by the Likert scale and has direct implications for training design.

### Parent Interaction Observations (Tool 2)

Table 5 presents mean scores for the 8 parent interaction competencies. The overall mean was 4.75 (SD 0.20; very high). All teachers (10/10, 100%) scored in the very high range across all 8 criteria. The highest-ranked criteria were empathetic family treatment (mean 4.92, SD 0.28; criterion 5), meeting preparation (mean 4.88, SD 0.33; criterion 3), and cultural respect (mean 4.88, SD 0.33; criterion 8). Clarity of structured communication (criterion 4; mean 4.48, SD 0.51) and IEP-aligned guidance (criterion 6; mean 4.52, SD 0.51) were the lowest-ranked criteria, both still classified as very high, representing the relative lower bound of an otherwise uniformly strong parent interaction profile.

**Table 5.** Mean scores and SDs for parent interaction competencies—tool 2.

Criterion number	Competency area	Score (1-5), mean (SD)^a^	Rank	Rating
1	Building relationships with parents based on mutual trust and cooperation	4.76 (0.44)	4	Very high
2	Listening to parents and involving them in decisions about child safety, health, and education	4.76 (0.44)	4	Very high
3	Adequate preparation for parent meetings and effective time management	4.88 (0.33)	2	Very high
4	Clarity and professionalism in delivering useful information and responding to questions	4.48 (0.51)	8	Very high
5	Treating all families fairly and empathetically, with sensitivity to their psychological context	4.92 (0.28)	1	Very high
6	Providing guidance based on the child’s individualized academic and behavioral plan	4.52 (0.51)	7	Very high
7	Effective communication using positive body language and a professional demeanor	4.76 (0.44)	4	Very high
8	Respect for Emirati cultural values and Islamic principles at all times	4.88 (0.33)	2	Very high

a Overall mean 4.75 (SD 0.20; very high).

### Qualitative Findings: Parent Interaction

Table 6 presents behavioral themes from parent meeting observations.

Trust building, cultural respect, and preparedness were pervasive across all participants. The use of Modern Standard Arabic, respectful Islamic greetings, and professional attire aligned with UAE norms were consistent across observations. Regarding communication clarity (criterion 4), evaluators noted that 40% (4/10) of the teachers across multiple sessions showed difficulty organizing complex verbal information in a sequential, parent-accessible manner when presenting multicomponent student progress data. This was a recurring qualitative theme that was consistent with the lowest quantitative ranking for this criterion.

**Table 6.** Key behavioral observations during parent meetings—thematic summary.

Criterion number	Competency area	Key observed practices
1	Trust and rapport	Demonstrated warm, approachable demeanor and created an atmosphere of comfort and confidence for parents.
2	Active listening and shared decision-making	Showed strong listening skills, responded constructively to parental concerns, and involved families in discussions about child development.
3	Preparation and time management	Arrived prepared with individualized plans and materials and communicated the meeting structure clearly.
4	Clarity of communication	The information was accurate and aligned with child needs; occasional inconsistencies in verbal organization were observed across cases.
5	Fairness, empathy, and family sensitivity	Treated all families with fairness and calm reassurance and was sensitive to the emotional state of parents.
6	IEP^a^-aligned guidance	Offered concrete, actionable guidance drawn from the student’s academic and behavioral framework and linked recommendations to home and school contexts.
7	Professional communication and body language	Maintained professional posture, eye contact, and tone; nonverbal cues complemented verbal explanations.
8	Cultural and religious sensitivity	Used Modern Standard Arabic and respectful greetings, avoided culturally inappropriate behaviors, and their professional appearance aligned with UAE^b^ norms.

a IEP: individualized education plan.

b UAE: United Arab Emirates.

## Discussion

### Principal Findings

This program-specific evaluation found that assistant teachers trained through 1 UAE diploma program demonstrated consistently high professional competence across classroom and parent interaction domains. Overall classroom means were 4.56 (SD 0.19; phase 1) and 4.55 (SD 0.23; phase 2), and the parent interaction mean was 4.75 (SD 0.20), all classified as very high ratings. Two criteria—daily living skill support and cultural and Islamic adherence—reached the maximum score in both phases. Safety, hygiene, and structured parent communication occupied the lower end of the performance distribution, classified as high, high, and very high, respectively. These findings are best understood as an initial evaluation of one specific professional preparation pathway; they should not be interpreted as representative of UAE assistant teachers generally.

### Measurement Considerations: SD of 0.00, Interrater Reliability, and Score Interpretation

The repeated findings of an SD of 0.00 across multiple criteria and evaluator sessions merit more than a brief acknowledgment. At least 3 interpretations must be considered. First, these values may reflect genuine ceiling-level uniformity for behaviors that the training program explicitly and systematically cultivated—particularly cultural adherence and daily living skill support—where the program’s clear behavioral expectations may have produced genuine compliance across all assessed teachers. Second, the pattern of an SD of 0.00 may reflect observer-related score compression: evaluators aware of the program’s cultural expectations may have rated cultural and routine behaviors at ceiling as a matter of professional expectation rather than independent behavioral discrimination. This is a form of observer-related scoring bias that cannot be excluded in the absence of formal interrater reliability testing. Third, the rubric itself may have limited sensitivity at the high end of the performance scale, producing floor effects at ceiling that compress discriminant variance regardless of actual performance differences.

The absence of formal interrater reliability statistics (ICC or κ) is the most significant methodological limitation of this study. Because quantitative scores were the product of 3 evaluators whose ratings were averaged rather than independently verified for agreement, it is not possible to determine with confidence whether high means reflect genuine uniformity of performance or shared evaluator assumptions. The consensus-based discrepancy resolution used in this study reduced obvious discrepancies but did not quantify the extent of agreement. Future evaluations must incorporate ICC or weighted κ statistics to establish measurement robustness. These findings should be interpreted as indicative rather than confirmatory, acknowledging this uncertainty.

Regarding the reactivity of being observed, even in unannounced observations, the presence of an expert evaluator may have elevated performance, particularly in culturally salient domains where social conformity expectations are strong. This form of observer-related reactivity is well documented in educational observational research [14] and may have contributed to the ceiling-level scores observed for cultural adherence criteria across all participants.

### Criterion 6 (Health and Nutrition; Phase 2): A Domain-Specific Finding

The substantially elevated SD for criterion 6 in phase 2 (1.15 vs 0.46 in phase 1) requires specific interpretation rather than treatment as a general measurement irregularity. This criterion uniquely combines applied nutritional knowledge with real-time classroom communication skills—a domain that may be more susceptible to individual variation in prior professional experience, health literacy background, and deployment context than procedurally anchored criteria such as transition monitoring or cultural adherence. The wide variability in phase 2 suggests that, among the 10 participants, some had developed sophisticated practical nutrition communication skills whereas others had not despite similar training exposure. This heterogeneity has specific implications: health and nutrition content in future training cohorts may benefit from differentiated instruction or competency-based checkpoints to ensure baseline consistency rather than uniform delivery.

### Performance Stability and Training Program Evidence

The near-identical overall means across the 2-month interval are consistent with the view that the diploma program produces competencies that are retained rather than decaying over short-term posttraining periods, as predicted by models of practice-based professional learning [15]. However, this stability in aggregate scores must be interpreted cautiously given the measurement limitations noted above, and it does not confirm that the program is responsible for observed competencies as no pretraining comparison data are available. Evidence-based professional development models integrating autism-specific content with coaching and peer learning are associated with the most robust competency gains [16,17], and the apparent stability of this program’s outcomes is encouraging as a preliminary finding pending more rigorous investigation.

### CRP: Theory and Practice

The consistent ceiling-level scores for cultural and Islamic value adherence across both observation phases and both evaluation domains provide behavioral evidence for the theoretical proposition by Gay [8] that cultural competence, when systematically embedded in teacher preparation, is reliably acquirable. The observed behaviors—Modern Standard Arabic instruction, modest attire, Islamic greeting conventions, and faith-integrated classroom routines—are precisely the behavioral dimensions of CRP that Ladson-Billings [9] identifies as foundational to culturally inclusive practice. Massouti et al [10] document that UAE inclusive education policy explicitly mandates these practices, suggesting that the evaluated diploma program successfully operationalized national policy expectations at the classroom behavioral level. The contribution of this finding for international readers lies in demonstrating that a culturally specific form of CRP can be formally assessed through observational rubrics, offering a methodological model for evaluating cultural competence in other non-Western inclusive education contexts [18].

### Safety, Hygiene, and Reactive-to-Proactive Training Design

The consistent high rating classification for safety and hygiene criteria across both phases, combined with qualitative findings documenting reactive rather than proactively integrated safety behaviors, points to a specific training gap: not in awareness but in procedural habituation. This distinction matters for training design: awareness-level interventions (information delivery and safety briefings) are insufficient if the goal is automatic, routine-embedded safety practice. There is evidence from analogous professional development contexts suggesting that behavioral rehearsal, scenario-based simulation, and routine-embedding protocols are more effective than didactic instruction for achieving procedural automaticity [19]. The uniform SD of 0.00 for safety items also raises the possibility that the current rubric may not discriminate adequately between reactive and proactive safety integration, suggesting that future instrument development should include behavioral indicators specifically targeting routine embeddedness.

### Parent Communication and IEP Translation

Communication clarity (mean 4.48, SD 0.51; very high; lowest ranked) and IEP-aligned guidance (mean 4.52, SD 0.51; very high; second-lowest ranked) occupied the lower end of the parent interaction profile. These criteria are conceptually distinct from the empathy and cultural responsiveness in which assistant teachers excelled: translating technical IEP data into accessible, sequential verbal guidance for parents requires meta-cognitive and expressive language competencies that are not routinely developed through general training. Structured communication tools—including scripted meeting frameworks, standardized IEP progress summary templates, and bilingual communication resources—represent evidence-supported interventions for this domain [20]. Mahmood et al [7] emphasize that administrative support structures also shape family engagement quality, suggesting that training-level interventions should be complemented by institutional frameworks that provide assistant teachers with preparation time, parent communication templates, and supervisory guidance.

### Scope and Generalizability

It bears repeating that these findings are derived from 10 assistant teachers drawn from a single diploma training program operating within a specific institutional partnership. The conclusions that can legitimately be drawn are as follows: (1) within this program, assistant teachers demonstrated high and stable competency profiles; (2) the evaluated program appears to successfully develop cultural competence and daily living skill support to ceiling level; and (3) safety, hygiene, and structured parent communication represent areas for targeted training improvement within this program. Claims about UAE assistant teacher performance broadly or about national policy implications exceed what this evidence can support and have been deliberately moderated in this study.

### Recommendations

The following practice-based recommendations are offered for this program’s future development:

- Develop safety and hygiene training modules incorporating behavioral rehearsal, scenario-based simulation, and routine-embedding protocols to promote proactive rather than reactive safety integration.
- Implement structured parent communication training using scripted meeting frameworks, standardized IEP progress summary templates, and bilingual communication resources complemented by institutional preparation time structures.
- Incorporate formal interrater reliability assessments (ICC or weighted κ) into all future observational evaluation protocols to strengthen measurement rigor.
- Revise the observational rubric to include behavioral indicators that discriminate between reactive and proactive safety integration, addressing the possible ceiling and sensitivity limitation identified.
- Consider differentiated instruction or competency checkpoints for health and nutrition content to address the interindividual heterogeneity identified in phase 2.

### Limitations

This study has several important limitations. First, the sample (N=10) drawn from a single diploma program substantially restricts generalizability; the findings describe this program’s graduates and should not be extrapolated to UAE assistant teachers broadly. Second, formal interrater reliability (ICC or κ) was not computed; the consensus-based resolution method used is insufficient to confirm score stability, and this materially limits confidence in individual criterion means. Third, the repeated SD values of 0.00 may reflect observer-related score compression, reactivity to observation, or limited rubric sensitivity rather than true performance uniformity. Fourth, the absence of pretraining comparison data means that no causal claims about training program effects can be made. Fifth, the study did not assess child developmental outcomes, precluding any inference about the relationship between teacher competence and student progress. Sixth, the 2-month observational window is insufficient to characterize longer-term performance trajectories.

### Future Research Directions

Future studies should (1) replicate this evaluation with larger multi-institutional samples across different UAE emirates to enable comparative analysis and normative data development, (2) incorporate formal ICC or weighted κ statistics and pretest-posttest training designs to strengthen methodological rigor, (3) examine the relationship between assistant teacher competency profiles and child developmental outcomes, (4) investigate the specific mechanisms through which health and nutrition knowledge variability (criterion 6) develops differentially across training cohorts, and (5) evaluate the effectiveness of the targeted training interventions recommended above through controlled or quasi-experimental designs.

### Conclusions

This program evaluation provides preliminary evidence that assistant teachers trained through 1 UAE diploma program demonstrate consistently high professional competence across classroom and parent interaction domains, with particular strengths in culturally responsive practice and daily living skill support. Two practice-level gaps—proactive safety and hygiene integration and structured IEP-linked parent communication—emerged as priority areas for training improvement within this program.

These findings should be understood as preliminary and program specific. They suggest that structured, culturally grounded diploma program training can support high professional performance in assistant teachers working with children with ASD in the UAE context, but replication with larger, multi-institutional samples incorporating formal interrater reliability measures is required before broader claims can be made. The measurement limitations of this study—particularly the absence of ICC statistics and the interpretive uncertainty associated with repeated SD values of 0.00—must be addressed in future evaluations.

## Abbreviations

ASD: autism spectrum disorder
CRP: culturally responsive pedagogy
ICC: intraclass correlation coefficient
IEP: individualized education plan
UAE: United Arab Emirates
